# IP-10 (CXCL10) Can Trigger Emergence of Dormant Breast Cancer Cells in a Metastatic Liver Microenvironment

**DOI:** 10.3389/fonc.2021.676135

**Published:** 2021-05-27

**Authors:** Amanda M. Clark, Haley L. Heusey, Linda G. Griffith, Douglas. A. Lauffenburger, Alan Wells

**Affiliations:** ^1^ Department of Pathology, University of Pittsburgh, Pittsburgh, PA, United States; ^2^ Pittsburgh VA Medical Center, VA Pittsburgh Healthcare System, Pittsburgh, PA, United States; ^3^ Hillman Cancer Center, University of Pittsburgh, Pittsburgh, PA, United States; ^4^ Department of Biological Engineering, Massachusetts Institute of Technology, Cambridge, MA, United States; ^5^ McGowan Institute for Regenerative Medicine, University of Pittsburgh, Pittsburgh, PA, United States

**Keywords:** metastasis, tumor dormancy, tumor emergence, IP-10, CXCL10, breast cancer dormancy, organ-on-a-chip, microphysiological system

## Abstract

Metastatic breast cancer remains a largely incurable and fatal disease with liver involvement bearing the worst prognosis. The danger is compounded by a subset of disseminated tumor cells that may lie dormant for years to decades before re-emerging as clinically detectable metastases. Pathophysiological signals can drive these tumor cells to emerge. Prior studies indicated CXCR3 ligands as being the predominant signals synergistically and significantly unregulated during inflammation in the gut-liver axis. Of the CXCR3 ligands, IP-10 (CXCL10) was the most abundant, correlated significantly with shortened survival of human breast cancer patients with metastatic disease and was highest in those with triple negative (TNBC) disease. Using a complex *ex vivo* all-human liver microphysiological (MPS) model of dormant-emergent metastatic progression, CXCR3 ligands were found to be elevated in actively growing populations of metastatic TNBC breast cancer cells whereas they remained similar to the tumor-free hepatic niche in those with dormant breast cancer cells. Subsequent stimulation of dormant breast cancer cells in the *ex vivo* metastatic liver MPS model with IP-10 triggered their emergence in a dose-dependent manner. Emergence was indicated to occur indirectly possibly *via* activation of the resident liver cells in the surrounding metastatic microenvironment, as stimulation of breast cancer cells with exogenous IP-10 did not significantly change their migratory, invasive or proliferative behavior. The findings reveal that IP-10 is capable of triggering the emergence of dormant breast cancer cells within the liver metastatic niche and identifies the IP-10/CXCR3 as a candidate targetable pathway for rational approaches aimed at maintaining dormancy.

## Introduction

Metastatic breast cancer remains a largely incurable and fatal disease. Advances in our abilities to remove and treat primary tumors have not yet translated into sustained success against metastatic disease. Recurrence occurs for ~20-30% of women diagnosed with invasive breast cancer (1). In particular, triple-negative breast cancer (TNBC) is a salient example with 25% of patients succumbing to recurrence within 5 years of their diagnosis (2).

The process of metastasis begins with cells within the primary tumor undergoing a cancer-associated epithelial to mesenchymal transition. This enables motility to disseminate into the circulation followed by extravasation into and colonization of distant organs *via* a partial reversion back to a more epithelial phenotype (3). Outgrowth into overt metastasis then occurs *via* another transition to a more mesenchymal phenotype (4). Strikingly, tumor cells can disseminate even at the earliest stages of primary tumor development (<5 mm) (5–7) and colonize ectopic sites as dormant cells or micro-nodules for years to decades before re-emerging into clinically detectable metastases (8). The signals that drive emergence represent targets for new rationale approaches to prevent metastatic recurrence yet the specific signals and associated mechanisms remain largely unknown.

Herein, we aimed to further our understanding into the developing but still uncertain picture of metastatic recurrence. For our investigations, we focused on the liver metastases as the liver is a major site of metastasis for breast cancer (9, 10) with its clinically evident involvement correlating most poorly with patient survival (11).

It is well-established that inflammatory signals, immune cells, and stromal components are involved in driving the outgrowth of dormant cells (12–18). Regarding the liver, we have shown that non-parenchymal cells of the liver (*e.g.* liver sinusoidal endothelial cells, Kupffer cells, hepatic stellate cells and tissue lymphocytes) can alter cell number and signaling of breast cancer cells, and when activated secrete factors that promote phenotypic changes indicative of emergence (13, 16, 17, 19–21). These studies focused on the role of the local microenvironment, but a hitherto underappreciated area is the involvement of inflammation from distant uninvolved organs.

The gut is the most pertinent distant organ that interacts with the liver as the portal circulation provides most of its blood supply. Dysregulation of the gut can result in increased translocation of bacterial products (*e.g*. lipopolysaccharide (LPS)) and other inflammatory signals. Although the liver routinely handles varying levels of bacterial toxins and inflammatory challenges from the gut, higher loads may overwhelm this homeostatic functioning and lead to overt inflammation, activation of resident liver non-parenchymal cells, and the potential stimulation of dormant tumor cells leading to emergence. Disruption of gut homeostasis has been shown to modulate cancer initiation, progression and dissemination (*e.g.* breast, pancreatic, liver, ovarian, prostate), and drug efficacy (22–24); but the role of gut-derived factors in metastatic disease remains to be determined.

Previously we have established and validated an all-human liver microphysiological system (MPS) – an *ex vivo* 3D organ-on-a-chip microfluidic model of the liver – that recreates metastatic breast cancer that is physiologically reflective of the human situation (19–21). It comprises an all-human biologically replete Liver MPS composed of a human donor-matched hepatic cells (hepatocytes and non-parenchymal cells). Using this *ex vivo* liver metastasis model we showed that quiescent dormant cells in the liver could be stimulated to re-emerge upon exposure to inflammatory products of gut inflammation [*e.g.* LPS/EGF (19)]. This correlated with our 2D data wherein activated non-parenchymal cells produced signals that promote phenotypic changes in breast cancer consistent with emergence (13, 16, 17). Additional insights were provided by a similar but more complex platform that supports interacting human liver and gut modules. The study by Chen et al. (25), found that under pathophysiological systemic inflammatory conditions, a significant non-linear modulation of signaling responses was observed, particularly the CXCR3 ligands (MIG/CXCL9, IP-10/CXCL10, and I-TAC/CXCL11). Together these data compelled us to investigate the possible role of CXCR3 ligands in driving emergence.

Our investigations revealed a significant correlation between the CXCR3 ligand, IP-10, with the TNBC subtype and shortened survival of breast cancer patients with metastatic disease. Using the cellular complex *ex vivo* liver MPS, IP-10 was then found to stimulate the emergence of dormant metastatic breast cancer cells in a dose-dependent manner and that the effect occurs *via* indirect mechanisms.

## Materials and Methods

### Reagents and Cell Sources

Donor matched human hepatocytes and non-parenchymal cells were isolated from excess pathological liver specimens. Patient donors included both males and females with no discernable differences among the genders or donors observed (19). Hepatic niche function and health was unaffected by patient donor background, the presence of breast cancer cells or treatments, and was maintained throughout the experiments (**Supplemental Figure 1**). The cells were obtained from 5 different donors through the Liver Tissue Cell Distribution System, Pittsburgh, Pennsylvania, and funded by NIH Contract # HHSN275201700005C. The liver specimens were provided as separate isolations of hepatocytes and non-parenchymal cells. The latter cells were further purified *via* Percoll gradients as previously reported (26).

The TNBC breast cancer cell line, MDA-MB-231, was purchased from ATCC and transfected with red fluorescent protein (RFP) as described previously (27). The cell line was maintained in RPMI 1640 supplemented with 10% heat-inactivated fetal bovine serum (FBS), 25 IU/mL penicillin and 25 IU/mL streptomycin (Gibco).

### *Ex Vivo* Metastatic Liver MPS

The *ex vivo* hepatic MPS (Legacy LiverChip^®^ by CNBio Innovations Ltd.) was assembled, seeded and maintained as previously described (19, 26). The functioning and bioengineering behind the MPS (*e.g.* physiological mimicry, fluid flow and oxygenation *etc.*) are also been explained in detail elsewhere (28, 29). Briefly, hepatocytes and non-parenchymal cells were seeded onto scaffolds coated with 1% rat-tail collagen type I (BD Biosciences) at a 1:1 ratio (6x10^5^ cells/scaffold) in William’s E Medium (WE; Life Technologies) supplemented with the Hepatocyte Thawing and Plating Supplement Pack (Life Technologies). Cells were cultured overnight and then the medium was changed to WE supplemented with the Hepatocyte Maintenance Supplement Pack (Life Technologies). After allowing the hepatic tissue to form, MDA-MB-231 cells expressing RFP (500 cells/scaffold) were introduced on day 3. Applicable cultures were treated with 1 µM doxorubicin (APP Pharmaceuticals LLC) on day 7 to 10 and then stimulated on day 13 to 15. The stimulus of 0.5, 1.0 or 5.0 ng/mL IP-10 (PeproTech) was administered in the presence or absence of 50 nM AMG-487 (Tocris). On day 15, scaffolds were removed and fixed in 2% paraformaldehyde at 4°C for 1 hour.

### Cell Enumeration and Morphology

Cancer cells within the scaffolds were imaged and cancer burden enumerated as previously described (19). Cell morphology for each cell was calculated based on the width to length ratio and determined by manually tracing and measuring pixel units of the midpoint width and cell length using ImageJ functions determining (16). Imaging instrumentation were provided by and performed at the Center for Biological Imaging, University of Pittsburgh.

### Proliferation Assay

MDA-MB-231 cells were seeded at a density of 1x10^4^ cells/well in 24-well plates containing 12 mm coverslips. After 24 hours, cultures were changed to quiescent medium (serum-free RPMI). After an additional 24 hours, cells were treated with 20 ng/mL IP-10, 50 nM AMG-487, 1 µg/mL LPS (Millipore Sigma) + 20 ng/mL EGF (Corning) in serum-free RPMI or HepM plus 10 µM EdU for 4 hours and then fixed with 2% paraformaldehyde at 4°C for 1 hour. Active proliferation was determined using the Click-iT PLUS EdU Alexa Fluor 488 Imaging Kit (Life Technologies) and detected according to the manufacturer’s instructions. Coverslips were mounted onto slides and imaged using the Nikon A1 microscope fitted with a 20x objective (Center for Biological Imaging, University of Pittsburgh).

### Dormancy Assay

Dormancy was mimicked *in vitro* as reported by Albrengues et al. (30). Briefly, a 96-well plate was coated with 50 µL growth factor reduced Matrigel^®^ (Corning) and incubated at 37°C for 30 minutes. MDA-MB-231 cells (2x10^3^) were re-suspended in RPMI containing 1% FBS and 2% Matrigel^®^ and seeded into each well. After 24 hours, medium was changed to RPMI containing 0.1% FBS, HepM or either medium plus 4 ng/mL IP-10, 50 nM AMG-487, IP-10/AMG-487, 1 µg/mL LPS + 20 ng/mL EGF or 5 ng/mL TGF-β, then cultured out to and imaged on day 5.

### Migration Assay

MDA-MB-231 cells were seeded at a density of 7.5x10^4^ cells/well in 24-well plates. After 24 hours, cultures were changed to quiescent media (0.1% dialyzed FBS; dFBS) overnight. Each well was scratched and then the media was changed to serum-free RPMI, HepM or either medium containing 1, 150 or 500 ng/mL IP-10, 1 µg/mL LPS + 20 ng/mL EGF or 100 ng/mL VEGF. Images were taken at 0, 4, 6 and 8 hours. The scratch width was measured using ImageJ 1.50i software and presented as percent change compared to 0 hours for each scratch.

### Invasion Assay

Transwell inserts, 12-well sized with 0.8 µm pores, were coated with 20% growth factor reduced Matrigel^®^ in serum-free RPMI and incubated at 37°C for at least 2 hours in order to solidify. MDA-MB-231 cells were re-suspended at a density of 1x10^5^ cells/mL in either in serum-free RPMI alone, HepM or either medium containing 20 ng/mL IP-10 or 10% FBS. In to each applicable transwell insert, 1x10^4^ cells were added to the apical of transwell inserts and serum-free RPMI, HepM or either medium containing IP-10 or FBS was added to the basolateral compartment. After 48 hours, cultures were fixed with 2% paraformaldehyde in PBS at 4°C for 1 hour. The cells on the upper surface of Matrigel^®^ were removed using a cotton swab. Inserts were then stained with 0.5% crystal violet in dH_2_O at room temperature for 10-15 minutes, washed thoroughly with dH_2_O and allowed to dry overnight prior to imaging. The color was then extracted from the inserts with a 2% SDS solution and the OD_550_ was determined using a spectrometer.

### Breast Cancer Datasets

Expression and survival data on females with Stage IV breast cancer were sourced from the TCGA dataset (n = 19 patients; sourced from the Human Protein Atlas version 19.3, http://www.proteinatlas.org). Patients were identified and selected based on being female and diagnosed with Stage IV breast cancer. Expression data based on breast cancer grade (n = 1832 patients) and hormone receptor status (n = 1903 patients) were obtained from the collated Curtis et al. (31), and Rueda et al. (32), datasets [European Genotype-Phenotype Archive under Accession number EGAS00000000083; sourced from cBioPortal v3.6.11 (33, 34)]. Patients were identified and selected based on their status being known for all estrogen, progesterone and HER-2 receptors. Two patients were removed due to missing estrogen receptor status.

### Statistical Analysis

All graphs were generated and statistical analyses were performed using GraphPad Prism version 9 (GraphPad Software Inc). The Mann-Whitney test was used to assess the *ex vivo* metastatic liver MPS data. Kaplan-Meyer survival curves were evaluated with the Gehan-Breslow-Wilcoxon test. The Mann-Whitney test was used to assess expression differences between TNBC and other breast cancer subtypes. Two-way ANOVA was used to assess migration data, while Student t-tests were used for invasion, proliferation and outgrowth data. Significance was set to *p*-value < 0.05.

## Results

### CXCR3 Ligands Are Associated With Tumor Outgrowth

**Figure 1:**
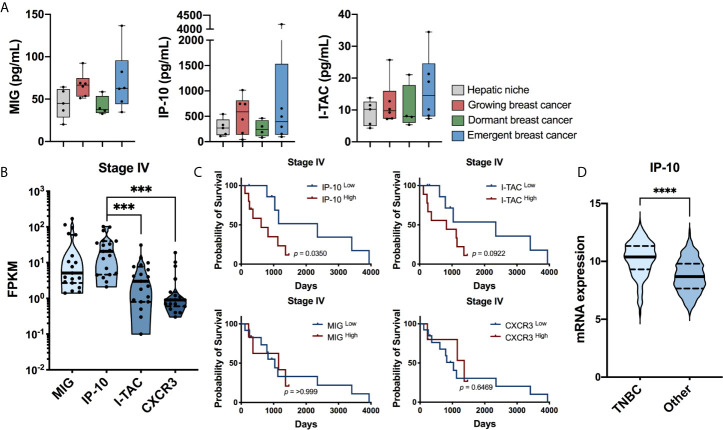
IP-10 is associated with metastatic progression**. (A)** Secreted protein levels of MIG, IP-10 and I-TAC in an *ex vivo* liver MPS for the hepatic niche alone, with growing breast cancer cells, with dormancy breast cancer cells or with emergent breast cancer cells determined using Luminex technology (median with quartiles and range; Mann-Whitney test; n = 4-6 donors). *Ex vivo* data sourced from: Clark et al. (19). **(B)** RNA expression levels human patients with in stage IV metastatic breast cancer (log10 transformation, median with quartiles and range, Wilcoxon matched-pairs signed rank test, *** *p* < 0.001). **(C)** Survival of the aforementioned patients based on high and low expression of IP-10, I-TAC, MIG and CXCR3 (Kaplan-Meyer survival analyzed with Gehan-Breslow-Wilcoxon test). **(B,C)** Human TCGA breast cancer data sourced from: Human Protein Atlas version 19.3 (http://www.proteinatlas.org; n = 19 patients). **(D)** mRNA expression levels of IP-10 in patients with TNBC compared to all other subtypes of breast cancer (median with quartiles and range; Mann-Whitney test, ****p < 0.0001). The human breast cancer dataset (31, 32) containing the hormone status data was sourced from cBioPortal v3.6.11 (33, 34) (n = 1903 patients).

Based upon the significant increase in CXCR3 ligands observed by Chen et al. (25) using a gut-liver MPS, further analysis was performed on previously published data, which modeled dormant-emergent metastatic progression within the Liver MPS (19) (**Supplementary Materials**). Within the prior study three different metastatic states were recreated: i) growing (Hep/NPC plus MDA-MB-231; a mix of actively proliferating and non-proliferating cancer cells), dormant (Hep/NPC plus MDA-MB-231 then doxorubicin; non-proliferating cancer cells) and emergent (Hep/NPC plus MDA-MB-231, then doxorubicin followed by LPS/EGF; reawakened proliferating cancer cells). Analysis of the effluent using multiplex protein immunoassays revealed that CXCR3 ligands were elevated in metastatic niches on day 15 of culture with actively growing populations of MDA-MB-231 cells (growing and emergent), whereas they remained similar to the tumor-free hepatic niche in those with dormant MDA-MB-231 cells (**Figure 1A**). While all three CXCR3 ligands were elevated in niches with actively growing tumor populations, IP-10 (CXCL10; growing 2.2-fold, emergent 1.5-fold) was present at markedly higher absolute levels than that of MIG (CXCL9; growing 1.5-fold, emergent 1.4-fold) and I-TAC (CXCL11; emergent 1.4-fold).

To observe if a connection exists between CXCR3 and its ligands with metastatic disease, their expression levels in patients with breast cancer were assessed (Human Protein Atlas version 19.3, http://www.proteinatlas.org). In those with stage IV disease, IP-10 was not only found to be the predominant ligand present but high expression was also associated with significantly lower survival (**Figures 1B,C**). Furthermore, IP-10 was the most abundant CXCR3 ligand in the *ex vivo* metastatic liver MPS (**Figure 1A**), stage IV patients (**Figure 1B**) as well as the study by Chen et al. (25), Additional analysis of data by Curtis et al. (31), and Rueda et al. (32), found IP-10 to be increased significantly with increasing tumor grade (**Supplementary Figure 2A**). Regarding breast cancer subtypes, it was most abundant in those with TNBC (**Figure 1D**), while lowest in ER+ (estrogen receptor) and ER/PR+ (progesterone receptor) cancer (**Supplemental Figure 2B**). Taken together, these suggest a possible role of IP-10 in metastatic breast cancer progression.

### IP-10 Promotes Emergence of Dormant Breast Cancer Cells

**Figure 2:**
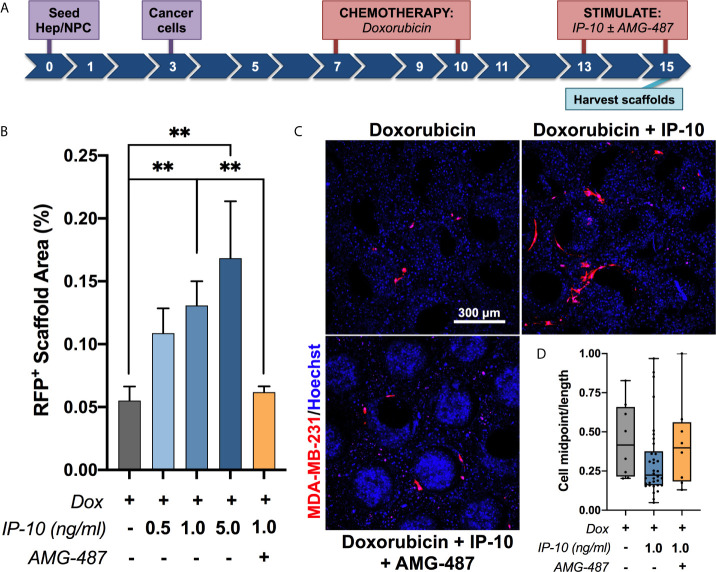
IP-10 promotes the emergence of dormant breast cancer cells in an *ex vivo* hepatic niche. **(A)** Experimental timeline schematic. **(B, C)** Quantification and representative images of the outgrowth of dormant MDA-MB-231 cells following stimulation in the presence or absence of an inhibitor (AMG-487) for 48 hours on day 15 (mean ± SEM; Mann-Whitney test, **p < 0.01; n = 2-5 donors). Blue – DAPI; Red – MDA-MB-231 RFP^+^ cells. **(D)** Quantification of the cell midpoint-to-length ratio of MDA-MB-231 RFP^+^ cells within the representative images depicted in **(C)** (median with quartiles and range).

In order to assess if IP-10 was involved in promoting emergence of dormant cells, our established *ex vivo* metastatic liver MPS method for modeling dormant-emergent progression (19, 26) was employed and modified as per **Figure 2A**. Briefly, MDA-MB-231 cells (expressing RFP) were seeded into the hepatic niche, wherein a subpopulation spontaneously colonize and attain a dormant phenotype (19, 20). To reflect the human situation, the metastatic hepatic niche was then treated with proliferation-targeting chemotherapy (doxorubicin) after which only the dormant MDA-MB-231 cells survive (19, 20). Emergence was then observed by measuring tumor burden after exposing the dormant MDA-MB-231 cells to the stimulus of IP-10 in the presence or absence of an inhibitor of IP-10 (AMG-487). The doses of IP-10 were based around the levels observed in the average levels observed in the effluent of the emergent group presented in **Figure 1A** (1 ng/mL) with additional doses chosen either side of the value (0.5 ng/mL and 5 ng/mL). Exposure of the dormant cells to IP-10 resulted in significant outgrowth of MDA-MB-231 cells and occurred in a dose-dependent manner (**Figures 2B, C**). Further, addition of AMG-487 significantly abrogated the outgrowth effect of IP-10. The MDA-MB-231 cells stimulated with IP-10 also appeared more elongated compared to the control (**Figure 2C**) and was reflected by a reduced midpoint-to-length ratio (**Figure 2D**) suggesting a possible partial reversion to a more mesenchymal phenotype.

### Breast Cancer Cell Proliferation Remained Unaltered by Direct Stimulation With IP-10

**Figure 3:**
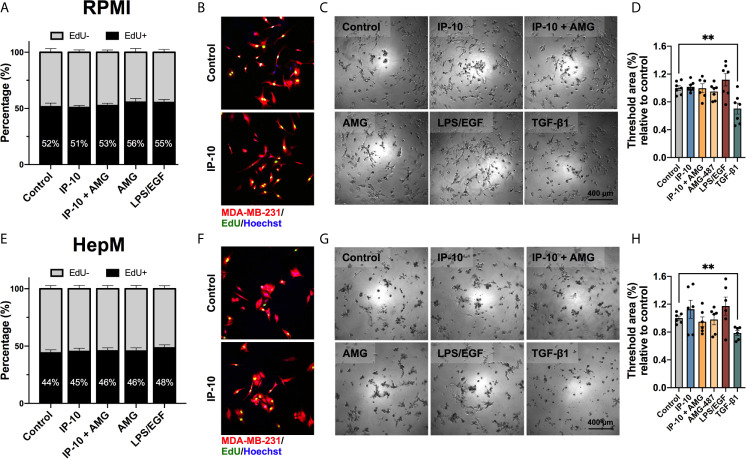
The proliferative activity of breast cancer cells is not directly affected by IP-10. MDA-MB-231 cells cultured in either **(A–D)** RPMI or **(E–H)** HepM medium. **(A, E)** EdU assay for 4 hours to assess proliferation (mean ± SEM; Mann-Whitney test; n = 4). **(B F)** Representative images of EdU assay. **(C, G)** Representative images of a dormancy Matrigel ^®^ outgrowth assay after 5 days in culture. **(D, H)** Quantified outgrowth (mean ± SEM; Student t-test, **p < 0.01; n = 3-4).

To determine if the impact of IP-10 on dormant breast cancer cells occurs directly or indirectly, we assessed tumor proliferation following exposure to proportionate levels of IP-10 used in the *ex vivo* metastatic liver MPS. MDA-MB-231 cells were plated at a low cell density to mimic *ex vivo* ratios. The influence of media was also mitigated by performing experiments in both the media for routine tumor cell culturing (RPMI) and in the *ex vivo* metastatic liver MPS (HepM). The proportion of cells actively proliferating was determined by quantifying the number of breast cancer cells that were positive for EdU. IP-10 stimulation was not associated with a significant increase in proliferation in either culture medium (**Figures 3A, B, E, F**). Overall, HepM medium was associated with lower proliferation compared to cells in RPMI. LPS/EGF was associated with increased proliferation compared to control in both mediums, however it was not significant.

Dormancy was then mimicked *in vitro* by embedding MDA-MB-231 cells in Matrigel^®^. Once again, IP-10 did not significantly impact the outgrowth of MDA-MB-231 cells in RPMI (**Figures 3C, D**). Outgrowth trends were seen for both IP-10 and LPS/EGF in HepM media and the latter in RPMI, however, a significant alteration in MDA-MB-231 cell behavior was only observed in the presence of TGF-β1 (negative control) (**Figures 3C, D, G, H**). The data suggests that levels of IP-10 relative to that experienced in the *ex vivo* metastatic liver MPS studies did not affect the proliferation capacity of MDA-MB-231 cells *in vitro*.

### Breast Cancer Cell Migration and Invasion Remained Unaltered by Direct Stimulation With IP-10

**Figure 4:**
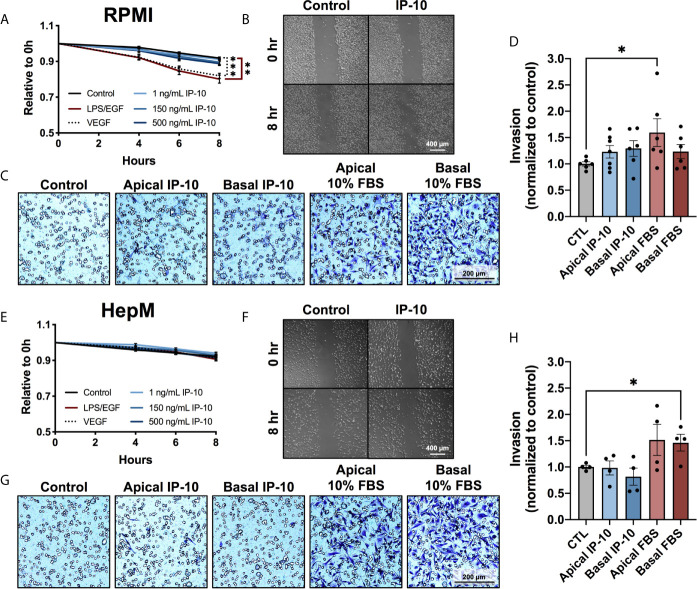
The migratory and invasive properties of breast cancer cells are not directly affected by IP-10. MDA-MB-231 cells cultured in either **(A–D)** RPMI or **(E–G)** HepM medium. **(A, E)** Scratch migration assay measured over 8 hours (mean ± SEM; Two-way ANOVA with Dunnett’s multiple comparisons test, **p < 0.01, ***p < 0.001; n = 3). **(B, F)** Representative images of the scratch migration assay at 8 hours. **(C, G)** Representative images of the invasion assay at 48 hours. **(D, H)** Quantification of invading cells (mean ± SEM; Student t-test, *p < 0.05; n=2-3).

As MDA-MB-231 cells were observed to transition to a more mesenchymal phenotype in the *ex vivo* metastatic MPS following stimulation with IP-10, the migratory and invasive propensities were investigated. The ability of IP-10 to promote migration was assessed through scratch assays in both RPMI and HepM. A slight increase in the migratory propensity of tumor cells in the presence of IP-10 was observed compared to control in RPMI, though there was no observable difference between the various doses which differed up to 500-fold (**Figures 4A, B**). However, LPS/EGF and VEGF (positive control) were associated with significant migration after 8 hours. Meanwhile, the tumor cells did not migrate in the HepM media even in the presence of the positive control (**Figures 4E, F**).

Invasive capabilities of MDA-MB-231 cells were investigated using transwells coated with Matrigel^®^. Invasion was promoted by the positive control of 10% FBS, while once again IP-10 only trended with a slight increase (**Figures 4C, D, G, H**). Similarly to the proliferation investigations, IP-10 relative to that experienced in the *ex vivo* metastatic MPS studies did not affect the migratory or invasive properties of MDA-MB-231 cells *in vitro*.

## Discussion

Metastasis remains a largely incurable disease and is the major cause of breast cancer-related mortality, with half of the disseminated disease emerging clinically 5 or more years after a seeming cure of the primary tumor (35). Delayed emergence is a result of clinically silent tumor cells which lay as dormant (non-proliferating) cells or nodules in the metastatic site for months to years to decades before suddenly emerging as lethal outgrowths (8, 14, 15). Discerning the operative molecular signals that drive this emergence is key to developing rational approaches to prevent recurrence.

Our model posited that dormant breast cancer cells in the secondary organ (*i.e.* the liver) re-emerge upon exposure to inflammatory cues from a distant, uninvolved organ (*i.e.* the gut) whose homeostasis has been disrupted. Dysregulation of the gut has been linked to the initiation, progression and dissemination of numerous cancers (22–24), however the role of gut-derived factors in metastatic disease remains to be determined. Recent data from an interacting *ex vivo* gut-liver MPS mimicking systemic inflammation identified CXCR3 ligands in particular as being synergistically up-regulated. The receptor itself, CXCR3 is involved both directly and indirectly in tumor progression by regulating tumor proliferation, migration, invasion, chemotaxis and immunity (36). Thus this finding prompted us to investigate if these ligands were involved in regulating metastatic progression of dormant breast cancer cells in the liver.

Our investigations identified a possible role for one CXCR3 ligand in particular, IP-10 (CXCL10), in driving the growth of metastatic breast cancer cells. Analysis of our *ex vivo* metastatic liver MPS – a liver only version of the gut-liver MPS – for CXCR3 ligands revealed increased levels of IP-10 in metastatic niches with actively growing MDA-MB-231 cells more so than MIG (CXCL9) or I-TAC (CXCL11). This *ex vivo* finding was supported by clinical data wherein high expression levels of IP-10 significantly correlated with worse survival in patients with stage IV breast cancer as well as increasing tumor grade. The former is also noted in numerous other cancer types, including melanoma (37), colorectal carcinoma (38), hepatocellular carcinoma (39), prostate cancer (40), and lung adenocarcinoma (41). Furthermore, IP-10 showed highest expression in those with TNBC disease and was the most abundant CXCR3 ligand observed in all three settings (*e.g. ex vivo* inflamed gut-liver MPS, *ex vivo* metastatic liver MPS and stage IV breast cancer patients). As all three ligands bind to and activate the same receptor, CXCR3, we focused on the chemokine with highest levels, IP-10.

With respect to metastatic progression, prior studies have demonstrated that IP-10 can promote dissemination and colonization of numerous cancer types (39, 42–47). However, its effect upon dormant tumor cells post-colonization remains largely unknown. Using our *ex vivo* metastatic liver MPS model, dormant MDA-MB-231 cells exposed to IP-10 were observed to emerge and outgrow. The effect of which was abrogated by AMG-487, CXCR3 antagonist. The importance of IP-10 derived from the metastatic microenvironment is supported by Lee et al. (43), who demonstrated that metastatic melanoma tumor burden was reduced in *IP10-/-* mice compared to wild type on day 14 but not day 7. IP-10 has also been found to be expressed in the normal liver tissue surrounding metastatic colorectal nodules in both mice and patient specimens (38). Our result is consistent with a prior report from Pradelli et al. (48) wherein CXCR3 and its ligands appeared to stimulate the expansion of the osteosarcoma lung metastatic foci in later stages. Furthermore, studies targeting CXCR3 in murine models of breast and melanoma observed inhibitory effects were specifically against tumor metastasis while the primary tumor mass was unaffected (49–51), indicating that CXCR3 has a role in promoting metastasis but not incidence. Combined these imply that host-derived IP-10 plays an important role in promoting the emergence of dormant metastatic cancer cells.

Interestingly, when MDA-MB-231 cells were exposed to IP-10 in the absence of the hepatic niche (*i.e.* alone), only minor changes in their proliferative, invasive and migratory behavior were observed. This was not necessarily unexpected given the intricate relationship that exists between dormant cells and their surrounding metastatic microenvironment. The absence of a significant effect by exogenously applied IP-10 implies that emergence is likely triggered in the hepatic niche *via* an indirect mechanism. Almost all cells within the metastatic microenvironment, including tumor, immune, stromal and endothelial cells express CXCR3 and are capable of secreting IP-10 (52, 53). This complexity likely accounts for the outgrowth observed in the *ex vivo* metastatic liver MPS, which was not replicated in simpler 2D *in vitro* assays. Within the liver, non-parenchymal cells are both the primary source and most responsive liver cells to inflammatory cues (54). They are capable of altering cell number and signaling of breast cancer cells, and when activated secrete factors that promote phenotypic changes indicative of emergence (13, 16, 17, 19, 21, 28). Subsequently, it is possible that the mechanism by which IP-10 exerts its effect occurs through activation of non-parenchymal cells that then secrete additional factors that stimulate the outgrowth of dormant breast cancer cells.

Determining which cell type within the hepatic niche is triggered by IP-10 to promote the outgrowth of dormant breast cancer cells is an important next step in order to better understand the biology underpinning metastatic emergence and in pursuit of rationale approaches to prevent it. Examination of the literature points towards two specific non-parenchymal cell types – hepatic stellate cells or Kupffer cells/macrophages (43, 55). Hepatic stellate cells express CXCR3 and are responsive to IP-10 in the liver microenvironment (55), while a reciprocal interaction between tumor cells and macrophages at the metastatic site was observed to promote outgrowth (43). Studies elucidating the cell type(s) and signaling network involved are ongoing but lie beyond the scope of the current missive.

In summary, we aimed to identify pathophysiological signals that drive emergence in order to help define candidates whose activities could be targeted to prevent metastatic recurrence. Our studies revealed CXCR3 ligands to be elevated in actively growing populations of metastatic breast cancer cells in a liver microenvironment. In particular, IP-10 was present at much higher levels than MIG and I-TAC, was highest in those with TNBC disease, and its high expression also correlated significantly with shortened survival times in breast cancer patients with metastatic disease. Using an *ex vivo* model of liver metastasis, IP-10 was then found to stimulate the emergence of dormant metastatic breast cancer cells in a dose-dependent manner. However, direct stimulation of breast cancer cells with IP-10 did not significantly change their migratory, invasive or proliferative capacity suggesting that IP-10 acts indirectly *via* surrounding metastatic microenvironment to drive emergence. The findings further confirm that metastatic microenvironment is an integral regulator of dormancy, and highlight the importance of focusing on signals derived by the microenvironment as possible targets for therapeutic strategies.

## Data Availability Statement

The raw data supporting the conclusions of this article will be made available by the authors, without undue reservation.

## Ethics Statement

The human cells utilized were determined to be exempt (not human research or human research not engaged) by the University of Pittsburgh IRB and USAMRDC Human Research Protection Office (HRPO).

## Author Contributions

AC and AW developed the concept, designed experiments, interpreted data, and wrote the manuscript. AC and HH performed the experiments and generated data. LG and DL reviewed the manuscript and contributed to interpretations. AC, AW and DL provided financial support. All authors contributed to the article and approved the submitted version.

## Funding

This study was supported by grants from the National Institutes of Health (UH3TR000496, GM69668, GM63569, U01-CA215798), VA Merit Award program and US Department of Defense (W81XWH-19-1-0494).

## Conflict of Interest 

AW and LG declare a patent on the Legacy LiverChip^®^ now commercialized by CNBio Innovations Ltd. LG also declares consulting fees paid by Zyoxel Ltd. in 2012 but no current relationship.

The remaining authors declare that the research was conducted in the absence of any commercial or financial relationships that could be construed as a potential conflict of interest.

## Abbreviations

CXCR3, CXC chemokine receptor-3; EGF, epidermal growth factor; HepM, hepatocyte media; IP-10, interferon gamma inducible protein-10; I-TAC, interferon-inducible T-cell alpha chemoattractant; LPS, lipopolysaccharide; MPS, microphysiological system; MIG, monokine induced by gamma; RFP, red fluorescent protein; TNBC, triple negative breast cancer; VEGF, vascular endothelial growth factor.
