# Supplementary material for: IP-10 (CXCL10) Can Trigger Emergence of Dormant Breast Cancer Cells in a Metastatic Liver Microenvironment
Source: Front Oncol. 2021 May 27;11:676135. doi: 10.3389/fonc.2021.676135 (PMC8190328; doi:10.3389/fonc.2021.676135)
Supplement: Supplementary file 1 [file DataSheet_1.docx]

Supplementary Material

# Supplementary methods

**Dormant-emergent model of metastatic progression in the *ex vivo* liver MPS**

Methods were taken with minor modifications from Clark *et al.,* (19) A model of dormant-emergent metastatic breast cancer progression enabling exploration of biomarker signatures, Molecular & Cellular Proteomics (17:4) pp. 619-630. Copyright © 2018 (The American Society for Biochemistry and Molecular Biology, Inc.). DOI: 10.1074/mcp.RA117.000370.

Ex vivo *hepatic MPS*

For the culturing protocols, the steps outlined in the “*Ex vivo* metastatic liver MPS” methods section were followed until day 6 after which applicable cultures were treated as follows: i) Growing breast cancer – standard media changes every 2 days, ii) Dormant breast cancer – 1 µM doxorubicin (APP Pharmaceuticals LLC) on day 7 to 10 (72 hours) followed by standard media changes every 2 days, and iii) Emergent breast cancer - 1 µM doxorubicin on day 7 to 10 (72 hours), media changes on day 11 followed by stimulation on day 13 to 15 (48 hours) with a combination of 1 µg/ml LPS (Sigma-Aldrich) and 20 ng/ml mouse EGF (Corning).

*Multiplex immunoassays*

On day 15, effluent samples were obtained for each experimental group and the signaling profiles determined. A total of 101 cytokines, chemokines, and growth factors were assayed for using the Human Group 1 panel (27-plex), Human Chemokine Panel (40-plex), Cancer Panel 1 (16-plex) and Cancer Panel 2 (18-plex). Assays were completed according to the manufacturer’s instructions (BioRad Laboratories), with the exception that coupled beads, biotinylated detection antibodies, and streptavidin-phycoerythrin fluorescent reporters were diluted 2-fold*.* All analytes were assessed from a total volume of 50 µL neat, undiluted culture medium per multiplex panel. To attain measurements within the working range of the assay, samples were diluted 2-, 4-, 8-, 16-, 32- and 100-fold specifically for 10 cytokines. Standard and sample diluents consisted of WE media in the presence of 0.75% bovine serum albumin (Sigma-Aldrich).

All samples and multiplex immunoassay panels were run simultaneously to avoid confounding differences from day-to-day and operator variability*.* Prepared arrays were assessed by the 3D suspension array system (BioRad Laboratories) utilizing xMAP technology licensed by Luminex. Data were collected with xPONENT for FLEXMAP 3D software, version 4.2 (Luminex Corporation) and results analyzed initially in BioPlex Manager software version 6.1 (BioRad Laboratories)*.* Absolute concentrations were calculated from median fluorescence intensity values via calibration to 15-point standard series that implemented a 2-fold serial dilution.

*Analysis of signaling data*

The concentration of each analyte was quantified using the 5-parameter logistic model to attain optimal curve fits for standards. The weighted sum of squared errors was minimized using logistic regression analyses. Typically, the weights are set equal to the inverse variance, but for immunoassays, the high-response end of a curve approaches saturation of the detector thus variance is approximated more appropriately by a power function [variance = A(response)^B^] where A is a function of the magnitudes of the responses and 1.0 ≤ B ≤ 2.0 for immunoassays. Curve fitting techniques were completed in BioPlex Manager software version 6.1 (BioRad Laboratories).

**Clinical chemistry assays**

Assays for glucose (GLU), blood urea nitrogen (BUN), aspartate transaminase (AST), and alanine aminotransferase (ALT) were performed in the College of American Pathologists certified clinical laboratories in the University of Pittsburgh Medical Center (UPMC, Pittsburgh, PA, USA) in accordance with all governmental regulations.

# Supplementary Figures and Tables

## Supplementary Figures

**Supplementary Figure 1.** **Hepatic tissue survives and functions through 15 days in the *ex vivo* microphysiological system.** Hepatocyte injury was evaluated through aspartate aminotransferase (ALT) and alanine aminotransferase (AST) enzyme levels. Metabolism and catabolism were measured by determining glucose consumption from the media (100 mg/dL) and blood urea nitrogen levels, respectively. Clinical chemistry assays were used to evaluate the levels of each marker. (A) Levels of analytes were determined on day 3, 7 and 15 for the hepatic niche, and day 7 and 15 for the hepatic niche plus MDA-MB-231 cells. Statistical analyses were performed but no significance was determined (mean ± SEM; Mann-Whitney test; n = 3-4 donors). (B) On day 15 levels of the injury marker AST and glucose consumption were measured in various hepatic experimental groups, including the hepatic niche alone, plus MDA-MB-231 cells or with doxorubicin as well as IP-10. Statistical analyses were performed but no significance was determined (mean ± SEM; Kruskal-Wallis with Dunn’s multiple comparisons test; n = 3-4 donors)

**Supplementary Figure 2. IP-10 is associated with more aggressive grades and the TNBC breast cancer subtype.** (A) mRNA expression levels of MIG, IP-10, I-TAC and CXCR3 based on histological tumor grade (median with quartiles, range and turkey outliers; Kruskal-Wallis test with Dunn’s multiple comparisons test, ** *p* < 0.01**** *p* < 0.0001; n = 1832 patients). (B) mRNA expression levels of IP-10 in breast cancer subtypes based on hormone receptor status (median with quartiles; Kruskal-Wallis test with Dunn’s multiple comparisons test, **** *p* < 0.0001; n = 1902 patients). The human breast cancer dataset containing histological grades and hormone status (31, 32) was sourced from cBioPortal v3.6.11 (33, 34).
